# Hypervirulent *Clostridium difficile* Strains in Hospitalized Patients, Canada^1^

**DOI:** 10.3201/eid1604.091152

**Published:** 2010-04

**Authors:** Michael R. Mulvey, David A. Boyd, Denise Gravel, Jim Hutchinson, Sharon Kelly, Allison McGeer, Dorothy Moore, Andrew Simor, Kathryn N. Suh, Geoff Taylor, J. Scott Weese, Mark Miller, the Canadian Nosocomial Infection Surveillance Program

**Affiliations:** Public Health Agency of Canada, Winnipeg, Manitoba, Canada (M.R. Mulvey, D.A. Boyd); Public Health Agency of Canada, Ottawa, Ontario, Canada (D. Gravel); Health Sciences Centre, St. John’s, Newfoundland and Labrador, Canada (J. Hutchinson and S. Kelly); Mount Sinai Hospital, Toronto, Ontario, Canada (A. McGeer); Montreal Children's Hospital, Montreal, Quebec, Canada (D. Moore); Sunnybrook Health Science Centre, Toronto (A. Simor); The Ottawa Hospital, Ottawa (K.N. Suh); University of Alberta Hospital, Edmonton, Alberta, Canada (G. Taylor); University of Guelph, Geulph, Ontario, Canada (J.S. Weese); and Jewish General Hospital, Montreal (M. Miller)

**Keywords:** Clostridium difficile, molecular typing, hypervirulent, community-onset, bacteria, pulsed-field gel electrophoresis, hospitalized patients, Canada, dispatch

*Clostridium difficile* infections (CDIs) have increased in incidence and severity within the past decade in North America and Europe (*1*), in large part because of the emergence of the hypervirulent North American pulsed-field type 1 (NAP1/027/III) strains (*2*–*5*). Recently, interest has increased in the ribotype 078 strain. A 2007 North American study showed that ribotype 078 strains predominated in swine and cattle (83%–94% prevalence), but were rare in a group of hospitalized persons (4% prevalence) (*6*). However, in studies from Europe and the United States, 078/V strains were found at a prevalence ranging from 3% to 11% (*7*–*9*). In a subsequent study by the US group, analysis of the toxinotype V strains from humans and food animals showed that 83% of strains were either NAP7 or NAP8 (*10*). A Dutch group has recently shown that 078/V strains increased from 3% to 13% during February 2005–2008 and can be considered hypervirulent (*11*). Our study aimed to determine the incidence rate of infections attributed to hypervirulent NAP7/078/V and NAP8/078/V strains of *C. difficile* in hospitals in Canada.

## The Study

The Canadian Nosocomial Infection Surveillance Program is a collaborative effort between the Canadian Hospital Epidemiology Committee, a subcommittee of the Association of Medical Microbiology and Infectious Disease Canada, the Centre for Infectious Disease Prevention and Control, and the National Microbiology Laboratory of the Public Health Agency of Canada. The Canadian Nosocomial Infection Surveillance Program conducted prospective surveillance including collection of stool specimens from patients showing the presence of CDI during November 2004–April 2005 and during March and April in 2007 and 2008.

An infection was considered healthcare-associated CDI if the patient’s symptoms occurred at least 72 hours after hospital admission or if the symptoms resulted in readmission of a patient who had been hospitalized within the 2 months before the symptom onset date and who was not a resident in a long-term care facility or nursing home (*12*). An infection was considered community-onset CDI if the healthcare-associated definition was not met. Outcomes 30 days postinfection were recorded to capture severe cases, which were defined as infections in patients admitted to an intensive care unit, in patients who had undergone colectomy, or in patients who had died (*12*). Deaths were assessed by the Canadian Hospital Epidemiology Committee member and categorized into 3 groups: 1) death directly attributable to CDI, 2) death indirectly related to CDI by exacerbation of an existing disease condition, or 3) death not a result of CDI. The assessment was made from information obtained from medical charts, nurse logs, laboratory reports, and consultation with nursing and medical staff.

All stool specimens were cultured for *C. difficile,* and isolates were analyzed by PCR and pulsed-field gel electrophoresis (PFGE) at the National Microbiology Laboratory. PFGE, ribotyping, and toxinotyping were performed as described (*10*,*11*). MICs were determined by agar dilution or Etest. The primers used for PCR and sequencing are listed in Table 1. Macrorestriction patterns were analyzed with BioNumerics V4.5 (Applied Maths, Sint-Martens-Latem, Belgium).

**Table 1:** Primers used in study of hospitalized patients with *Clostridium difficile* infection, Canada, 2004–2008

Primer	Sequence (5′ → 3′)	Specificity
*tcd*3	TGCAATTATAAAAACATCTTTAAAC	*tcdC* PaLoc negative regulator
*tcd*4	TATATCTAATAAAAGGGAGATTG	
*cdtB*-F1	TGGACAGGAAGAATAATTCCTTC	*cdtB* binary toxin subunit B
*cdtB*-R1	TGCAACTAACGGATCTCTTGC	
E5	CTCAAAACTTTTTAACGAGTG	*ermB* erythromycin/clindamycin resistance
E6	CCTCCCGTTAAATAATAGATA	
*GyrAF*	TTGAAATAGCGGAAGAAATGA	*gyrA* DNA gyrase subunit A
*GyrAR*	TTGCAGCTGTAGGGAAATC	
*GyrBF*	GAAGGTCAAACTAAAACAAA	*gyrB* DNA gyrase subunit B
*GyrBR*	GGGCTCCATCTACATCG	

Fifteen NAP7 and 4 NAP8 patterns were identified from isolates obtained from 2,794 patients (overall prevalence 0.68%). Table 2 lists the patients and epidemiologic information, and the Figure shows the corresponding genomic fingerprint patterns. During the study period, the incidence rate increased as follows: 8/1,785 (0.5%) in 2004–2005; 5/638 (0.8%) in 2007; and 6/371 (1.6%) in 2008. Of the 19 patients identified, 14 were men with an average age of 70.8 years (not including 1 pediatric case), and 4 were women with an average age of 52.2 years; the overall average age was 61.5 years (Table 2). CDI was considered as community onset in 7 (37%) of 19 cases, and severe CDI was manifested in 3 (15.8%) case-patients (1 was healthcare-associated CDI and 2 were community-onset CDI). At 30 days postinfection for CDI, 26.3% of all patients had died, 1 death a direct result of CDI (5.3%), and 1 indirectly related; 10.6% of total deaths were attributable to CDI.

**Table 2:** Epidemiologic information from hospitalized patients with *Clostridium difficile* infection, Canada, 2004–2008*

Year and patient ID	Province	Age, y/sex	Source	Severe CDI†	Outcome‡
2004–2005					
O1-0059	Ontario	62/M	Healthcare-associated	No	Discharged
O2-0053	Ontario	35/M	Community-onset	No	Died-not attrib
O3-0042	Ontario	64/F	Community-onset	No	Discharged
Q1-0028	Quebec	66/M	Healthcare-associated	Yes	Died-attrib
H1-0040	Nova Scotia	70/M	Healthcare-associated	No	Discharged
S1-0054	Saskatchewan	72/M	Community-onset	No	Discharged
S1-0063	Saskatchewan	82/M	Community-onset	Yes	Discharged
O7-0121	Ontario	74/M	Healthcare-associated	No	Survived-hosp
2007					
O1-7-0011	Ontario	87/M	Community-onset	No	Survived-hosp
O4-7-0011	Ontario	82/M	Community-onset	Yes	Died-contrib
Q1-7-0017	Quebec	40/F	Healthcare-associated	No	Discharged
O8B-7-0002	Ontario	65/M	Healthcare-associated	No	Died-not attrib
Q5-7-0013	Quebec	71/M	Healthcare-associated	No	Discharged
2008					
B1-8-0052	British Columbia	44/F	Healthcare-associated	No	Discharged
B1-8-0059	British Columbia	73/M	Healthcare-associated	No	Discharged
A3-8-0022	Alberta	38/F	Community-onset	No	Discharged
O2B-8-0015	Ontario	75/F	Community-onset	No	Survived-hosp
Q1-8-0008	Quebec	81/M	Healthcare-associated	No	Died-not attrib
O5-8-0001	Ontario	2/M	Healthcare-associated	No	Discharged

*ID, identification; CDI, *Clostridium difficile* infection; Died-not attrib, death not attributable to CDI; Died-attrib, death directly attributable to CDI; Survived-hosp, patient survived but was still in a hospital at endpoint; Died-contrib, CDI indirectly contributed to death. †Required admission to intensive care unit due to CDI, received a colectomy, or died. ‡At 30 days after diagnosis of CDI.

**Figure:**
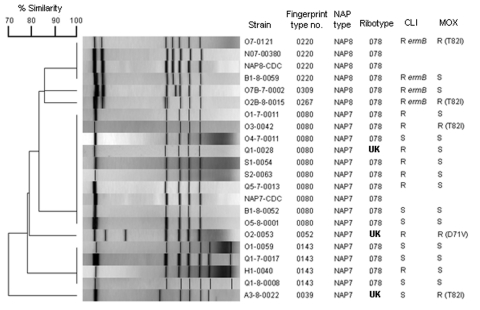
Dendrogram analysis of macrorestriction patterns (*Sma*I) of the NAP7 and NAP8 *Clostridium difficile* strains isolated from the patients listed in Table 2. *C. difficile* N07-00380 is a ribotype 078 control strain. *C. difficile* NAP7-CDC and NAP8-CDC control strains are toxinotype V. Isolates exhibiting high-level clindamycin resistance (>256 μg/mL) and harboring *ermB* are indicated. The amino acid change found in the gyrA protein is shown for the moxifloxacin-resistant strain antimicrobial drug–resistance mechanisms.

Sequence analysis of the *tcdC* gene showed that all strains carried a C184T transition that introduces a stop codon leading to a presumptive truncated protein of 61 residues, and a 39-bp deletion located downstream of the alternative stop codon. This *tcdC* variant has been previously described for toxinotype V strains (*13*). Sixteen of the isolates were ribotype 078 and 3 isolates had unknown ribotypes. All 2004/2005 and 2007 isolates were toxinotype V. The 2008 isolates were not toxinotyped. All 19 strains were susceptible to metronidazole and vancomycin. Seven isolates were susceptible to clindamycin (MIC <8 μg/mL) and 12 were resistant (6 had MICs = 8, 2 had MICs = 16, and 4 had MICs >256). Only the 4 latter strains carried *ermB* and all were NAP8. Fourteen isolates that were susceptible to moxifloxacin (MIC <8 μg/mL) had identical *gyrA* and *gyrB* quinolone-resistance–determining regions (QRDR) sequences to the genes in *C. difficile* 630 (GenBank accession no. AM180355). Five moxifloxacin-resistant isolates (MIC > 8 μg/mL) had no mutations in the *gyrB* QRDR but each had 1 mutation in the *gyrA* QRDR. One with MIC = 8 had an Asp71Val mutation; 3 with MIC = 16 and 1 with MIC >32 had a Thr82Ile mutation. These mutations have been previously described in moxifloxacin-resistant *C. difficile* (*14*).

## Conclusions

*C. difficile* NAP7 and NAP8/078/V strains are relatively rare in hospitalized patients with CDI in Canada, in contrast to their prevalence in Europe and the United States (*7*–*11*). However, incidence rates have tripled from 0.5% in 2004 to 1.6% in 2008 (p = 0.22). There was a high association with a community onset, although dataset was too small to statistically confirm that increased cases were more likely to be community onset; 2 (40%) of 5 deaths were attributable to CDI. Although the number of strains studied here was small, data are consistent with other studies that indicate a community association for NAP7 and NAP8/078/V strains (*9*–*11*). The prevalence of these strains in Canada may be higher than suggested here if they are a common cause of community-associated CDI, as studies have indicated (*10*,*11*). The role of animals in acquisition of NAP7 and NAP8/078/V strains was not evaluated because animal and food contact data were not available.

Molecular typing of *C. difficile* is typically performed by using ribotyping in Europe and PFGE/macrorestriction analysis in North America; both groups may use toxinotyping, which strictly looks at PaLoc variation. We showed a high correlation between NAP7, NAP8, ribotype 078, and toxinotype V strains by the 3 typing methods, which enabled results of separate studies to be compared. Furthermore, *tcdC* analysis provides an additional diagnostic tool for these strains because the gene has a 39-bp deletion and a C184T-transition in all isolates we studied.

Continued surveillance is warranted in humans, animals, and retail meat to determine whether NAP7 and 8/078/V strains will continue to emerge in patients hospitalized in Canada and to determine whether the sources of these infections are related to animals or food. Surveillance is especially important given that these strains appear to be hypervirulent as has been reported for NAP1/027/III strains (*11*).
